# A Focused *In Situ* Hybridization Screen Identifies Candidate Transcriptional Regulators of Thymic Epithelial Cell Development and Function

**DOI:** 10.1371/journal.pone.0026795

**Published:** 2011-11-07

**Authors:** Qiaozhi Wei, Brian G. Condie

**Affiliations:** Department of Genetics, University of Georgia, Athens, Georgia, United States of America; Oklahoma Medical Research Foundation, United States of America

## Abstract

**Background:**

Thymic epithelial cells (TECs) are necessary for normal T cell development. Currently, one transcription factor, Foxn1 is known to be necessary for the progression of fetal TEC differentiation. However, some aspects of fetal TEC differentiation occur in Foxn1 mutants, suggesting the existence of additional transcriptional regulators of TEC differentiation. The goal of this study was to identify some of the additional candidate transcription factors that may be involved in the specification and/or differentiation of TECs during fetal development.

**Methodology/Principal Findings:**

We identified candidate fetal TEC transcriptional regulators via data and text mining. From our data mining we selected the transcription factors *Foxg1*, *Isl1*, *Gata3*, *Nkx2-5*, *Nkx2-6* and *Sox2* for further studies. Whole mount *in situ* hybridizations confirmed the expression of these transcription factors within subdomains of the third pharyngeal pouch from E9.5–E10.5. By E11.5 days *Foxg1* and *Isl1* transcripts were the only mRNAs from this group of genes detected exclusively within the thymus domain of the third pouch. Based on this initial *in situ* hybridization analysis, we focused on defining the expression of *Foxg1* and *Isl1* during multiple stages of thymus development and TEC differentiation. We found that *Foxg1* and *Isl1* are specifically expressed in differentiating TECs during fetal and postnatal stages of thymus development. In addition, we found differential expression of *Islet1* and *Foxn1* within the fetal and postnatal TEC population.

**Conclusions/Significance:**

Our studies have identified two developmental transcription factors that are excellent candidate regulators of thymic epithelial cell specification and differentiation during fetal development. Our results suggest that *Foxg1* and *Isl1* may play a role in the regulation of TEC differentiation during fetal and postnatal stages. Our results also demonstrate heterogeneity of TECs marked by the differential expression of transcription factors, potentially providing new insights into the regulation of TEC differentiation.

## Introduction

Thymic epithelial cells (TECs) are a critical component of the thymic microenvironment. TECs are derived from the endoderm of the third pharyngeal pouch. Despite their essential role in thymus function, our current understanding of fetal TEC specification and differentiation is very limited. For example, we do not know which transcriptional regulators are necessary for the earliest specification of the thymus organ domain within the third pharyngeal pouch. In addition we have very limited knowledge about the transcription factors that regulate the differentiation and function of TECs during fetal and postnatal thymus development. Identifying the factors that regulate these key steps in the development of thymic epithelial cells is a key part of understanding the genetic pathways that regulate thymus organogenesis and function.

Our current knowledge regarding the earliest events in the specification of the parathyroid and thymus suggests that specification occurs early in third pouch development. Localized expression of *Gcm2* at E9.5 in the parathyroid domain and *Foxn1* at E11.25 in the thymus domain of the 3rd pharyngeal pouch marks the patterning of the pouch into the primordium of the two organs [1], [2]. However, it is clear that 3rd pouch patterning is well underway during pouch formation or shortly thereafter. In the case of the thymus, a grafting study showed 3rd pouch endoderm from E9.0 day old embryos was able to form a functional thymus when it was transplanted under the kidney capsule of an adult mouse [3]. This indicated that at E9.0 a developmental program is underway that is sufficient for the differentiation of a functional thymus from explants of 3rd pouch endoderm. Although the pouch graft result suggests that the thymus domain of the 3rd pouch is specified by E9.0, the only transcription factor known to be expressed specifically within the thymus primordium in the 3rd pouch is *Foxn1* which is first detected at E11.25 [2]. The gap in timing between the time of the 3rd pouch competency to form the thymus primordium in a graft and the time when *Foxn1* is first expressed suggests that additional transcription factors are acting within the pouch at times prior to *Foxn1* expression. These factors include the transcriptional regulators that activate Foxn1 within the thymus primoridium.

Previous studies have identified the transcription factors *Hoxa3*, *Pbx1*, *Tbx1*, *Pax1*, *Pax9*, *Six1* and *Eya1* as necessary for 3rd pouch development. All of these transcription factors, except for *Tbx1* are expressed throughout the 3^rd^ pouch at E10.5 prior to detectable *Foxn1* expression [1], [4], [5], [6], [7], [8], [9]. *Tbx1* is initially expressed throughout the 3rd pouch and becomes restricted to the presumptive parathyroid domain at E10.5 in the pouch endoderm [1], [4], [6], [10]. In the case of *Hoxa3*, *Eya1*, *Six1*, *Pax9*, *Tbx1* and *Pbx1* the homozygous mutants either fail to form the 3rd pouch or exhibit very severe early defects in the formation of both the thymus and parathyroid primordia [4], [5], [7], [9], [11], [12], [13], [14]. The very early and severe defects in pouch outgrowth and/or differentiation of these knockout mice are not informative about the role each gene may have in pouch patterning and/or later thymus or parathyroid differentiation.

A major goal of this screen was to identify candidate developmental transcription factors that play an important role in 3rd pharyngeal pouch endoderm development and/or the differentiation of fetal TECs into functional components of the thymus microenvironment. To enable the characterization of the genes identified in our screen we focused our screen on genes for which well-characterized knockout mice are available. In the case of Gata3, which we chose for detailed *in situ* hybridization analysis based on previous expression data, our genetic studies have shown that it is necessary for the normal development of the third pharyngeal pouch [15]. The third pouch degenerates in *Gata3* mutants at E12.5 days indicating an early role for this gene in the development of the pouch [15]. This result suggests that our approach is a viable way to identify new regulators of third pouch and/or TEC development.

## Results

### Data mining generated a short list of candidate transcription factors for detailed *in situ* hybridization analysis

A large amount of published and unpublished mouse developmental gene expression data is available in online databases [16], [17], [18], [19]. Although database *in situ* hybridization data are limited in terms of resolution, they can be used to suggest candidate genes for in depth characterization. We took advantage of this information to focus our *in situ* hybridization analysis to a short list of candidate transcription factors that were likely to be expressed in localized domains of the 3^rd^ pouch and/or thymic epithelium. For third pharyngeal pouch expression we visually screened data in the MGI/GXD and Emage *in situ* hybridization databases and examined published reports describing Cre recombinase expression patterns [16], [18], [20], [21]. Within these databases we focused on known developmental regulators that appeared to be expressed in the foregut endoderm or in the pharyngeal region. We also focused on transcription factors for which a conventional or conditional knockout mouse was readily available to us. Our data mining identified members of the Nk2/3, forkhead box, GATA binding protein, Sox/SRY-box containing and LIM/homeodomain families as candidates for genes expressed within the third pharyngeal pouch. Examination of E14.5 *in situ* hybridization of para-sagittal sections in the Genepaint database indicated that most of the genes we had identified as being expressed in the third pouch were also expressed within the thymus at E14.5. This suggested that these transcription factors were excellent candidates for genes involved in thymus development and differentiation. After additional literature mining, we selected *Nkx2-5*, *Nkx2-6*, *Foxg1*, *Islet1*, *Gata3* and *Sox2* for further analysis in this study. The screen of *in situ* database information allowed us to focus on performing a detailed characterization of a small group of genes rather than performing a less detailed large-scale screen.

### Regionalized expression patterns of transcription factors in the 3^rd^ pharyngeal pouch endoderm prior to onset of Foxn1 expression

Although pouch endoderm as early as E9.5 is capable of developing into a fully functional thymus when transplanted under the kidney capsule [3], the transcriptional regulators that lead to *Foxn1* expression within the thymus domain of the third pouch at E11.25 days are not known. One purpose of our study was to search for transcription factors with regionalized expression patterns in the third pharyngeal pouch endoderm between E9.5–E11.25. We analyzed the expression of our candidate genes in E9.5 and E10.5 somite stage matched wild type mouse embryos by *in situ* hybridization and 3D reconstruction, focusing on their expression in the pharyngeal pouches. By carefully staging embryos by somite counting we found that embryos at the same somite stage had comparable pouch morphologies as determined by 3D reconstruction of paraffin sections. By comparing the expression patterns of the genes we examined to the *Gcm2* expression pattern at E10.5 as a landmark for organ specific domains, we found a surprisingly diverse and complex combination of regionalized expression patterns in the third pouch endoderm.

### *Nkx2-5* and *Nkx2-6* are expressed in the ventral endoderm of the developing 3^rd^ pouch

Previous studies of *Nkx2-5* and *Nkx2-6* expression have detected expression of *Nkx2-5* in the pharyngeal endoderm and *Nkx2-6* in the pharyngeal pouch endoderm. However, these studies have not described the expression pattern of either gene in the 3rd pouch in detail [22], [23], [24], [25]. Examination of Cre recombinase expression patterns uncovered a published image indicating that *Nkx2-5* is expressed in a localized domain of the 3^rd^ pouch [10]. Furthermore, a recent study has shown that a small fraction of the EpCAM+ cells in the E14.5 day thymus are derived from a lineage that expressed *Nkx2-5* at some point in its history [26]. In addition, previous genetic studies have shown that pharyngeal endoderm development is very abnormal in *Nkx2-5*/*Nkx2-6* double mutants [27] demonstrating a role for the genes early in pharyngeal endoderm development. These previous studies suggested that both genes were excellent candidates for more detailed analysis.

**Figure 1:**
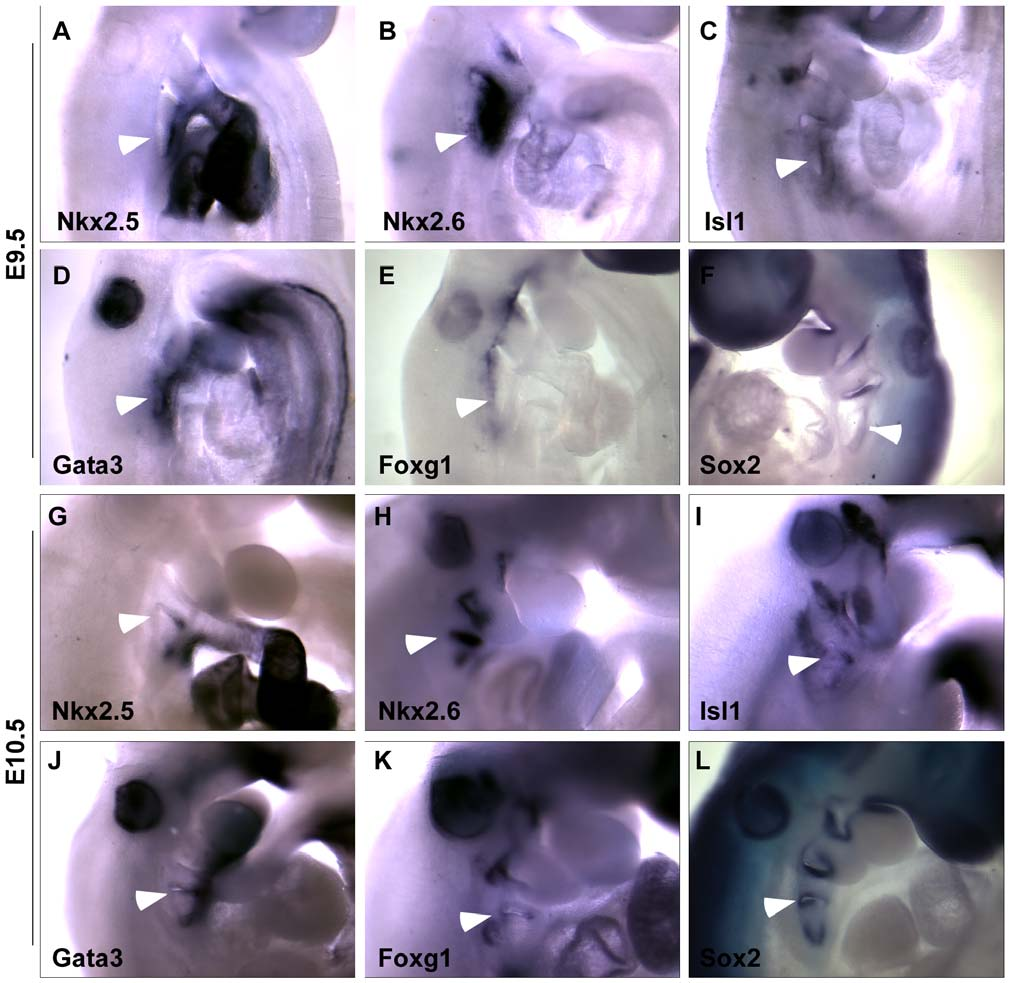
Expression of *Nkx2-5*, *Nkx2-6*, *Isl1*, *Gata3*, *Foxg1* and *Sox2* in the pharyngeal region at E9.5 (20–23 somites) and E10.5 (30–33 somites) as detected by whole-mount *in situ* hybridization. (A–F) *Nkx2-5*, *Nkx2-6*, *Isl1*, and *Gata3* are expressed in the developing 3^rd^ pouch at E9.5. (G–L) At E10.5, *Nkx2-5*, *Nkx2-6*, *Isl1*, *Gata3* and *Foxg1* are expressed in the ventral portion of the 3^rd^ pouch endoderm while *Sox2* is expressed in the dorsal portion of the 3^rd^ pouch. Arrowheads indicate the 3^rd^ pharyngeal pouch.

**Figure 2:**
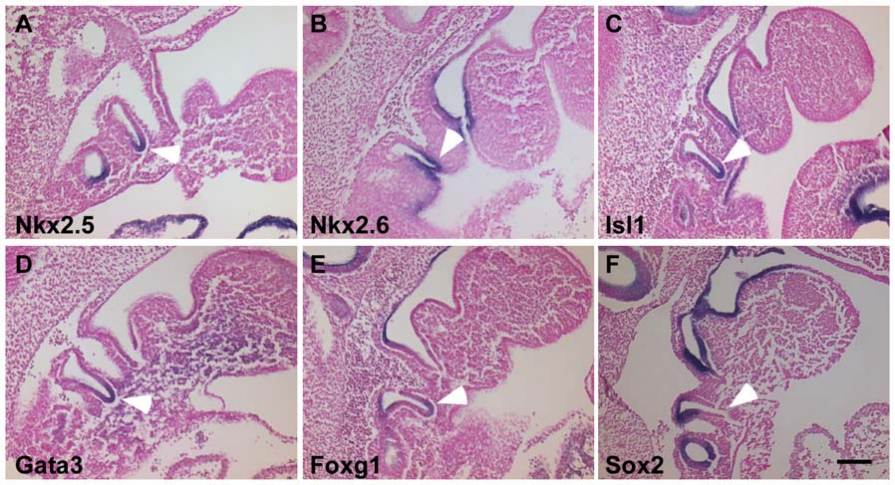
Regionalized expression patterns of *Nkx2-5*, *Nkx2-6*, *Isl1*, *Gata3*, *Foxg1* and *Sox2* in the 3rd pharyngeal pouch at E10.5 (30–33 somites). Parasagittal sections (10–14 µm) of embryos that were hybridized to the indicated probes are shown. Anterior is up and dorsal is left. Arrowheads indicate the 3rd pharyngeal pouch. All of the genes are expressed in the ventral 3^rd^ pouch except for *Sox2* (F). Scale bar represents 100 µm.

**Figure 3:**
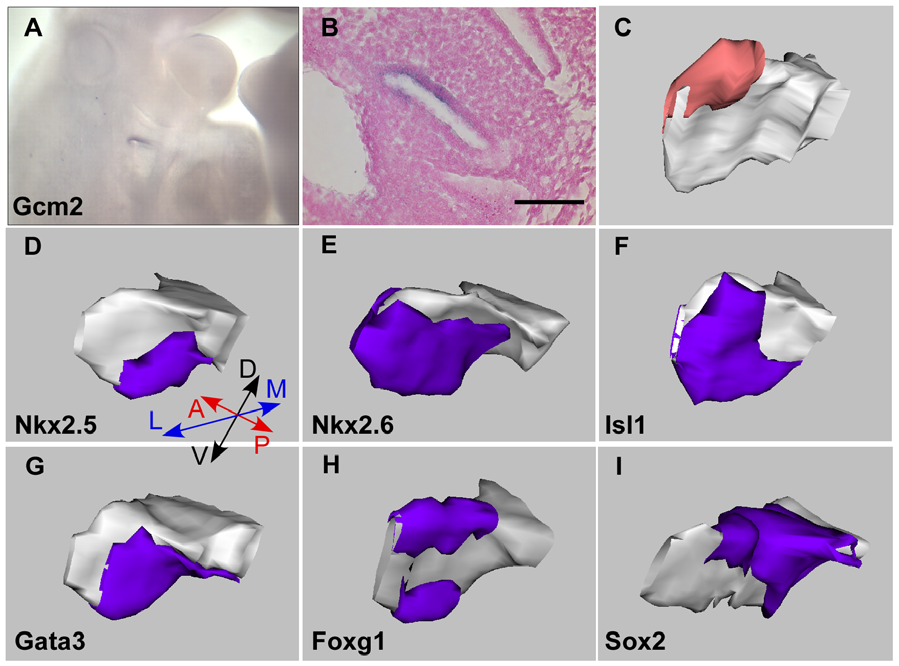
3D reconstructions of the 3^rd^ pouch reveal differentially regionalized expression patterns of *Nkx2-5*, *Nkx2-6*, *Isl1*, *Gata3*, *Foxg1* and *Sox2* at E10.5 days. (A–C) Expression pattern of *Gcm2* in the third pouch at E 10.5. Whole mount (A) and parasagittal sections (B) of E10.5 embryos hybridized with a *Gcm2* probe. (C) A left third pouch was reconstructed showing *Gcm2* expression in red. (D–I) 3D reconstructions of the left 3^rd^ pouch showing the expression of each gene in blue. Orientation of the reconstructed pouch is shown in (D). D, dorsal; V, ventral; A, anterior; P, posterior; L, lateral; M, medial. Scale bar represents 100 µm.

Our whole mount *in situ* hybridization analysis revealed that *Nkx2-5* and *Nkx2-6* are expressed in localized regions of the third pouch. At E9 days (20–23 somites) *Nkx2-5* is expressed on the ventral side of the forming third pharyngeal pouch endoderm (Figure 1A). In contrast, *Nkx2-6* is expressed in a much wider region covering a majority of the 3^rd^ pouch and its surrounding mesenchyme (Figure 1B). At E10 (30–33 somites), *Nkx2-5* transcripts become restricted to the ventral tip of the 3rd pouch while *Nkx2-6* expressing region extends more dorsally and laterally in the 3^rd^ pouch endoderm (Figure 1G, H and Figure 2A, B). The 3D reconstructions of sections from E10 embryos show that the *Nkx2-5* domain corresponds to a proximal ventral region that is about one third of the 3rd pouch endoderm and the *Nkx2-6* domain covers almost three fourths of the pouch and is localized in the ventral portion of the pouch (Figure 3D, E). The 3D reconstructions of the expression patterns revealed that the *Nkx2-5* expression domain is likely to be a subset of the *Nkx2-6* domain.

### *Islet 1* (*Isl1*) and *Gata3* are also expressed in localized domains of the ventral 3^rd^ pouch

Previous studies have detected *Isl1* expression in foregut endoderm and *Gata3* expression in the pharyngeal pouches [28], [29], [30]. *Isl1* expression has been well characterized in other endoderm derived organs such as the lung and pancreas but its expression in the pharyngeal endoderm has not been reported. Genetic studies have shown that *Isl1* expression is necessary for normal pharyngeal endoderm development [29]. In the case of *Gata3* very limited data (a single section) indicated that it is expressed in the thymus domain of the E10–E10.5 (30–35 somites) 3rd pouch [28]. In humans, mutations in *Gata3* are associated with a syndrome of hypoparathyroidism, sensorineural deafness and renal disease [31]. These genetic data provide additional support for a role for *Gata3* in development of 3^rd^ pouch derivatives.

Our analysis showed a complex pattern of expression for *Isl1* during pharyngeal pouch development. At the 20–23 somite stage, *Isl1* is expressed in the cells on the ventral side of the developing second and third pharyngeal pouches but on the dorsal portion of the first pouch (Figure 1C). The *Isl1* expression pattern in the 3rd pouch at this stage closely resembles that of *Nkx2-5* (Figure 1A, C). By the 30–33 somite stage, *Isl1* expression becomes clearly localized to the ventral posterior portion of both the 2^nd^ and 3^rd^ pouch but only a small dorsal part of the 1^st^ pouch. It is also expressed in the newly formed 4^th^ pouch (Figures 1I, 2C). By both section and 3D reconstruction comparisons, *Isl1* expression seems to largely overlap with *Nkx2-6* in the third pouch but with less expression on the anterior side and more on the posterior side of the third pouch. In contrast, *Nkx2-6* seems to be evenly expressed on both anterior and posterior sides of the third pouch, symmetrically labeling the whole ventral distal part of the third pouch at this stage (Figure 3E, F). Isl1 is also expressed in the ectoderm proximate to the 3^rd^ and 4^th^ pouch endoderm.

We detected *Gata3* expression in the cells of the ventral end of the forming 3^rd^ pharyngeal pouch at 20–23 somites (Figure 1D). This expression is similar to that of *Nkx2-5* and *Isl1* but extends more caudally. By 30–33 somites, its pharyngeal endoderm expression becomes specific to the ventral part of the 3^rd^ and 4^th^ pouch also mimicking that of *Nkx2-5* and *Isl1* (Figure 1G, I, J and Figure 2D). However, the *Gata3* expression domain covers a wider portion of the pouch endoderm than the *Nkx2-5* domain (Figure 3D, G). *Gata3* is also expressed in the arch mesenchyme ventral to the pharyngeal pouches.

### *Foxg1* is expressed in two discrete regions of the developing third pharyngeal pouch endoderm

*Foxg1* expression and function have been extremely well characterized in the CNS, where it is necessary for normal telencephalic development [32]. However, *Foxg1* expression in other tissues has not been examined in detail. Previous studies have shown that a knockin allele of *Foxg1*, designed to express Cre recombinase from *Foxg1* regulatory sequences, expresses Cre activity in the pharyngeal pouches [13], [33], [34]. An examination of *in situ* hybridization data in the Emage database confirmed RNA expression in the pharyngeal region. These results led us to examine *Foxg1* expression in the 3rd pharyngeal pouch in much greater detail.

We found that *Foxg1* is expressed in two domains of the 3rd pharyngeal pouch endoderm after pouch formation and prior to *Foxn1* activation. At 20–23 somites *Foxg1* mRNA is expressed in the foregut endoderm but is not detected in the forming 3^rd^ pouch (Figure 1E). As the 3^rd^ pouch grows (30–33 somites), *Foxg1* expression is detected at the ventral tip of the 3^rd^ pouch endoderm (Figure 1K). On sections of embryos at 30–33 somites hybridized to the *Foxg1* probe, we found two discrete regions of *Foxg1* expression in the 3^rd^ pouch endoderm, one on the ventral side of the pouch and another at the dorsal but proximal corner of the pouch (Figure 2E). Also, 3D reconstructions show that the *Foxg1* expression region on the ventral side of the 3^rd^ pouch is mostly overlapping with that of *Nkx2-5*, while the dorsal domain is similar to the *Sox2* domain but is distinct from the *Gcm2* domain (Figure 3H).

### *Sox2* is expressed in a novel subdomain of the 3rd pharyngeal pouch

Previously published data provided the rationale for examining the expression of *Sox2* in the 3rd pharyngeal pouch. It has been shown that *Sox2* is expressed in the foregut endoderm at E9.0 and there is very limited immunofluorescence data indicating that *Sox2* protein is expressed in the pharyngeal endoderm including pharyngeal pouch endoderm at E9.5 [35], [36]. However, no detailed information about *Sox2* expression in the 3^rd^ pouch is available. In addition, direct interactions between the *Sox2* and *Eya1* proteins have been reported recently [37] and *Eya1* function is known to be required for normal third pouch development [12], further supporting the need to carefully document the expression pattern of *Sox2* in the 3rd pouch.

Our analysis revealed a dynamic pattern of *Sox2* expression in the pharyngeal pouch. At 20–23 somites *Sox2* is only expressed in the first two pouches, but not in the forming third pouch endoderm (Figure 1F). By 30–33 somites *Sox2* expression is detected throughout the endoderm of pharyngeal pouches 1, 2 and 4 and in the dorsal 3^rd^ pharyngeal pouch, but is excluded from the ventral part of the third pharyngeal pouch (Figure 1L, 2F). This novel subdomain of *Sox2* expression in the 3^rd^ pouch partially overlaps with the *Gcm2* expression pattern but most of the *Sox2* transcripts are detected in a more proximal and posterior portion of the pouch (Figure 3I). We also performed *in situ* hybridizations with a Sox3 probe, but we did not detect Sox3 expression in the 3^rd^ pouch from E9–E10.5 (data not shown).

### Localized expression of *Foxg1* and *Isl1* within the thymus/parathyroid primordium at E11.5

The expression of *Nkx2-5*, *Nkx2-6*, *Isl1*, *Gata3* and *Foxg1* in the ventral portion of the third pharyngeal pouch suggested that these factors might be expressed in the thymic epithelial cells at later stages. To test this idea we examined the expression of these 5 transcription factors in E11.5 wild type embryos. In the E11.5 third pouch endoderm, the thymus domain can be identified by the expression of Foxn1 in the ventral posterior portion of the pouch, complimentary to the parathyroid domain which is marked by Gcm2 expression [2].

**Figure 4:**
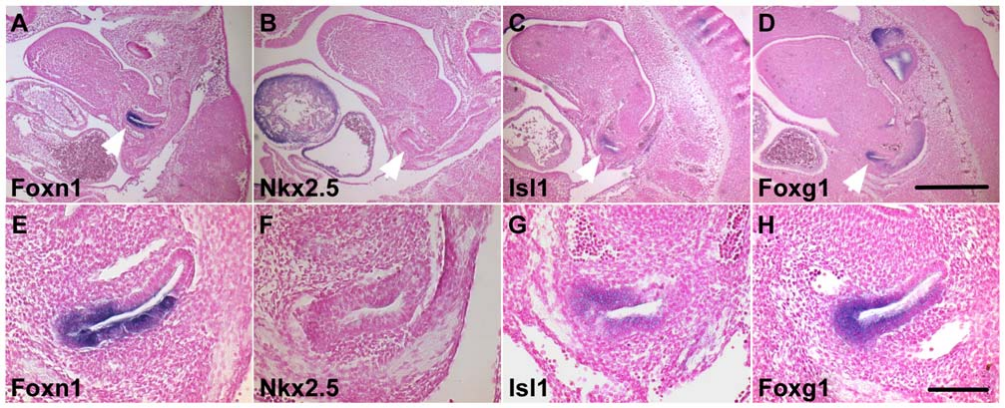
Continued expression of *Isl1* and *Foxg1* in the ventral third pouch endoderm/thymus rudiment at E11.5. Parasagittal sections of E11.5 embryos hybridized with *Foxn1* (A, E), *Nkx2-5* (B, F), *Isl1* (C, G) and *Foxg1* (D, H) probes. Ventral is on the left and anterior is up. Arrow heads in A–D indicate the third pouch. Scale bar represents 500 (A–D) and 100 µm (E–H).

Our *in situ* hybridization analysis showed that *Nkx2-5*, *Nkx2-6* and *Gata3* are not expressed within the thymus domain of the third pouch at E11.5. In fact, at this stage *Nkx2-5* and *Nkx2-6* are no longer expressed in any of the pharyngeal pouches (Figure 4B,F and data not shown). In the case of *Gata3* we observed a very dynamic expression pattern in the third pouch endoderm. Although *Gata3* is expressed in a ventral domain of the 3^rd^ pouch at E10.5, we found that it becomes expressed within the dorsal parathyroid domain that is marked by *Gcm2* expression at E11.5 [15].

In contrast, *Isl1* and *Foxg1* expression at E11.5 are each restricted to the thymus domain of the third pouch endoderm (Figure 4C, D, G, H). Strikingly, their expression in other pharyngeal endoderm structures is down-regulated by this time leading to localized expression within the thymus domain of the 3rd pouch (Figure 4C, D). Although *Foxg1* expression is still present in part of the second pouch at E11.5, the second pouch degenerates later in development and does not contribute to any organs or structures in rodents [38]. *Isl1* expression in the pharyngeal endoderm was found to be exclusively restricted to the thymus domain of the third pouch. Our results strongly suggest that *Isl1* and *Foxg1* are expressed in the early thymus domain of the pouch prior to *Foxn1* expression. Currently, the functional significance of the differential but overlapping expression of *Isl1* and *Foxg1* at E10.5 is not clear.

### *Isl1* and *Foxg1* continue to be expressed in TECs through late fetal and postnatal differentiation

To test whether *Isl1* and *Foxg1* are expressed in late fetal and postnatal thymic epithelial cells we performed double immunofluorescent antibody staining to detect FOXG1 or ISL1 protein co-expression with FOXN1 in wild type E16.5 embryos and in 2 and 4 week old postnatal thymus. Our results have revealed molecular heterogeneity in the expression of these developmental regulatory factors in TECs after the onset of *Foxn1* expression.

**Figure 5:**
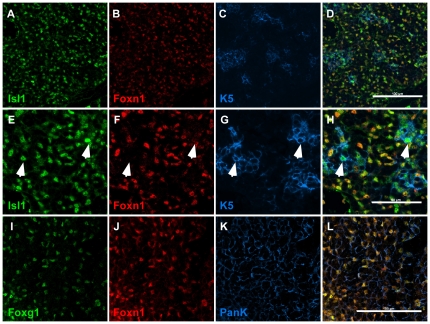
ISL1 and FOXG1 protein expression in the fetal thymus. Paraffin embedded or frozen transverse sections from E16.5 embryos stained with anti-ISL1, FOXN1 and KRT5 (K5, Keratin 5) antibodies (A–H), or anti-FOXG1, FOXN1 and Pan-cytokeratin antibodies (I–L). Arrows indicate ISL1 and KRT5 positive but FOXN1 negative cells. No signal was seen in no-primary controls for all antibodies. Scale bars represent 100 µm in A–D, I–L and 50 µm in E–H.

In sections of E16.5 fetal thymus, ISL1 expression was detected in all FOXN1-expressing thymic epithelial cells (Figure 5A–D and data not shown). Intriguingly we also detected ISL1 positive nuclei that exhibited no or very low levels of FOXN1 expression (Figure 5E–H). To confirm that these ISL1**^+^** FOXN1**^−^** cells were TECs, we co-stained the sections with an antibody to keratin 5 (KRT5) protein, which has been shown to be expressed in medullary thymic epithelial cells and in a subset of the cortical epithelial cells at late fetal and postnatal stages [39]. This analysis showed that the ISL1 positive but FOXN1-negative/low cells were clearly positive for KRT5, indicating they were TECs (Figure 5E–H). FOXN1-negative TECs have been described in previous studies [40], [41]. One study reported that about 20% of TECs do not express detectable FOXN1 protein as early as E13 with a similar number of FOXN1 negative TECs at E16 [40]. However, our results suggest that at E16.5 FOXN1-negative/low epithelial cells are quite rare.

In contrast, we found that FOXG1 expression in E16.5 thymus completely co-localizes with FOXN1 in TECs (Figure 5I–L). The antibody we used detected FOXG1 expression in the developing telencephalon and thymus in wild type embryos but resulted in no staining in the no-primary antibody control sections or tissues from a *Foxg1* null embryo (data not shown) [33]. Unfortunately, due to the fundamental differences in the staining procedures used for the ISL1 and FOXG1 antibodies, we were unable to perform co-localization of FOXG1 and ISL1 on the same thymus sections.

**Figure 6:**
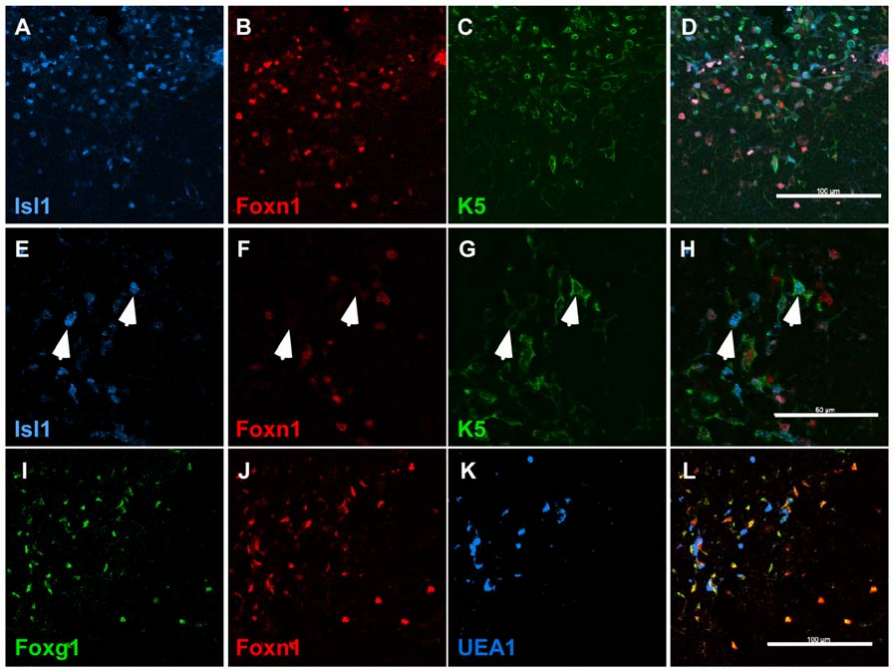
ISL1 and FOXG1 expression in postnatal thymus. Paraffin or frozen sections from 2–4 week old thymus stained with anti-ISL1, FOXN1 and KRT5 antibodies (A–H), and anti-FOXG1 and FOXN1 antibodies and the UEA1 lectin (I–L). Note the presence of ISL1 and KRT5 positive but FOXN1 negative cells indicated by arrows. Scale bars represent 100 µm in A–D, I–L and 50 µm in E–H.

In 2 and 4 week postnatal thymus, ISL1 and FOXG1 continue to be broadly expressed in most, if not all thymic epithelial cells (Figure 6A–D and data not shown). In contrast to E16.5, ISL1-positive but FOXN1-negative or low thymic epithelial cells are present at a much higher frequency at postnatal stages, and are almost exclusively found in the medulla as revealed by co-localization of KRT-5 and ISL1 staining (Figure 6E–H). This is consistent with the postnatal down-regulation of FOXN1 in the TECs [42]. Also, we see differential expression levels of FOXG1 and FOXN1 in the medullary region (Figure 6I–L). Our results are consistent with previous reports that *Foxn1* transcript and protein expression are at various levels in medullary thymic epithelial cells [43]. In addition, FOXN1^high^; ISL1^low^; FOXG1^low^ TECs and FOXN1^high^; ISL1^high^; FOXG1^high^ TECs were widely present at these stages (Figure 6).

## Discussion

Our focused *in situ* hybridization screen has revealed several transcription factors with novel localized expression patterns in the 3^rd^ pharyngeal pouch endoderm prior to activation of *Foxn1* expression. Our analysis documented localized expression of *Foxg1*, *Isl1*, *Gata3*, *Nkx2-5*, *Nkx2-6* and *Sox2* in the third pouch prior to the activation of *Foxn1* at approximately E11.25. In addition, we have shown that two of these factors, *Isl1* and *Foxg1*, continue to be expressed in E11.5 thymus primordium as well as in later fetal and postnatal TECs. Each of these transcription factors is known to have important functions in the patterning, development, and differentiation of other cell types and organs. In addition, most of the genes we examined are expressed in the thymus domain of the pouch prior to the activation of *Foxn1*. Our results suggest that these genes are excellent candidates for future genetic studies of their role in pouch specification, differentiation and survival and in early thymus organogenesis. In fact, we have recently reported that *Gata3* is required for the survival of the third pharyngeal pouch cells after E12.5 [15]. Another intriguing possibility is that the transcription factors we have studied may be involved in the appropriate activation of *Foxn1* expression within the thymus domain of the third pouch and/or the continued expression of *Foxn1* in the differentiating TECs.

Other groups have carried out large-scale screens to search for genes involved in thymic microenvironment development [44], [45], [46]. One study focused on stromal gene expression in the thymus in 4–8 week old mice [44]. This extensive study identified a number of genes as being expressed primarily in adult thymic stroma. Of particular interest, *Isl1* and *Foxg1* were found as highly expressed adult stromal transcription factors by this analysis. However this study did not examine gene expression at fetal or early postnatal stages, the developmental times we focused on for our analysis. Another study used *in situ* hybridization to examine the expression of several transcription factor families starting at E15.5 days. This study reported that Foxg1 expression was not detectable at E15.5 but was expressed at postnatal days 0 and 30. These authors reported expression of *Isl1* at E15.5, P0 and P30 but at very low levels. Unlike our analysis, the authors of this study did not examine gene expression in the early fetal stages, prior to the onset of TEC-thymocyte crosstalk [39] and during the earliest stages of TEC differentiation. Also, they also did not define the cell type in which *Foxg1* and *Isl1* were expressed [46]. A third gene expression screen focused on identifying genes preferentially expressed in the 3rd pharyngeal pouch versus 2nd pharyngeal arch [45]. This screen identified three 3rd pouch specific transcription factors, *Pax1*, *Gcm2* and *Mafb*. Since *Pax1* and *Gcm2* already had well-established roles in 3rd pouch development the authors focused on *Mafb* and have shown that it is expressed in thymic mesenchyme and that it plays a role in normal thymus development [45].

Our work has revealed previously unknown complex and dynamic gene expression patterns of *Foxg1*, *Isl1*, *Gata3*, *Nkx2-5*, *Nkx2-6* and *Sox2* within the 3^rd^ pharyngeal pouch from E9–E10.5. This degree of complexity is surprising because at this time the pouch is simply being subdivided into the thymus and parathyroid domains [2]. One possibility is that the early expression patterns reflect the specification of different thymic epithelial cell sub-lineages prior to Foxn1 expression. Consistent with this possibility, it has been reported recently that a subpopulation of Ep-CAM^+^ CD31^−^ PDGFRα^−^ TECs may be derived from an *Nkx2-5* expressing lineage [26].

It is also possible that some of the factors we have characterized are involved in *Foxn1* independent genetic pathways of TEC differentiation. Although *Foxn1* is necessary for the progression of TEC differentiation after E11.5, some aspects of TEC differentiation initiate in *Foxn1* mutants. An excellent example of a *Foxn1* independent aspect of TEC differentiation is the activation and fetal expression of *IL 7* in *Foxn1*−/− TECs [47]. In wild type embryos *IL7* expression is activated between E10.5 and E11.5 [47]. It is exclusively expressed in the 3rd pharyngeal pouch and is restricted to the thymus domain of the pouch [47]. IL7 plays a crucial role in thymocyte proliferation with IL7−/− individuals exhibiting greatly reduced thymocyte cell numbers at fetal and postnatal stages [48], [49]. Overall these results indicate that *IL7* activation and expression is a crucial part of TEC differentiation but that *IL7* expression does not require *Foxn1* function.

In addition, our analysis has demonstrated that *Isl1* and *Foxg1* are expressed in developing thymic epithelial cells through out thymus ontogeny. *Isl1* is expressed in the ventral third pouch endoderm as early as E9.5, while *Foxg1* can be detected in a smaller domain of the ventral third pouch endoderm at E10.5. By the time *Foxn1* expression is detectable, *Foxg1* and *Isl1* expression is localized within the thymus domain of the pouch and they are broadly co-expressed with *Foxn1* throughout fetal stages. Given that *Isl1* and *Foxg1* are expressed prior to *Foxn1* activation we infer that Foxn1 activity is not required for the activation of these two factors. Therefore, *Isl1* and *Foxg1* are good candidates for regulators of TEC differentiation pathways that are upstream or independent of Foxn1 function.

A previous study has suggested that at postnatal stages TECs can remain in a functional and differentiated state without the expression of Foxn1 [40]. The Foxn1- postnatal TECs are derived from Foxn1+ cells but continue to express CCL25 and DLL4 [41]. In this context it is intriguing that we detected ISL1 protein expression within many cells in the FOXN1^−^ medullary TEC population. This suggests that ISL1 may be involved in regulating the survival or function of these FOXN1^−^ medullary TECs. Our data suggest a novel heterogeneity in the expression of developmental transcriptional regulators among the postnatal medullary epithelial cells.

## Materials and Methods

### Whole-mount *in situ* hybridization and post hybridization sectioning

All work with mice conformed to the stipulations of the University of Georgia Institutional Animal Care and Use Committee. All of the work with mice in this study was reviewed and approved by the University of Georgia Institutional Animal Care and Use Committee. Swiss-Webster (Taconic) embryos were dissected in DEPC-PBST (DEPC treated phosphate buffered saline and 0.1% Tween 20) and somite number was determined. Embryos within a narrow range of somite stages were pooled in groups of 3–5 for processing. Therefore, we refer to individual embryos as being within a range of somite numbers since we cannot accurately count somites after the *in situ* hybridization. After fixation in 4% Paraformaldehyde at 4 C overnight, embryos underwent washes in PBST, 25%, 50% and 75% methanol in PBST and 100% methanol. Embryos then were stored in −20°C. The whole-mount *in situ* hybridizations were performed as previously described [9], [50]. After hybridization, the embryos were re-fixed in 4% PFA overnight, dehydrated in methanol and processed for paraffin embedding. 10 to 14 µm parasagittal sections were cut and counterstained with nuclear fast red (Sigma).

Digoxygenin (DIG)-labeled antisense RNA probes were synthesized using standard procedures. All probe templates were generated by PCR reactions using either mouse genomic DNA or cDNA clones as templates. In all cases, probe templates were carefully designed to not include highly conserved sequences to eliminate the possibility of cross hybridization. In the following primer sequences the lower case letters indicate the phage promoters. For *Nkx2-5*, a cDNA plasmid clone was generated by RT-PCR using E11-day mouse total RNA (Clontech). Primers were *Nkx2-5*-3-F: CTACG GCGTG GGTCT CAATG C and *Nkx2-5*-3-R: GCGTT AGCGC ACTCA CTTTA ATGG. The transcription template was generated by PCR using the SP6 and T7 promoter primers and using our *Nkx2-5* plasmid cDNA as template. The *Nkx2-6* probe template was amplified from mouse genomic DNA using primers: T7-*Nkx2-6*-F: taata cgact cacta tagg ACTGGTACTGGACGGCAAGC and SP6-*Nkx2-6*-R: attta ggtga cacta taga GCACAGCATCTACGTGGCTA. *Isl1* probe template was generated by PCR from an MGC cDNA (accession BC132263) using primers: *Isl1*F-T7: taata cgact cacta taggT CATCC GAGTG TGGTT TCAA and *Isl1*R-SP6: attta ggtga cacta tagaT GAATG TTCCT CATGC CTCA. *Foxg1* probe template was generated by PCR from an MGC cDNA (accession BC046958) using primers: *Foxg1*F: AGTTACAACGGGACCACGTC and *Foxg1*R-T3: aatta accct cacta aagg CCCCT GATTT TGATG TGTGA. The *Sox2* probe template was generated by PCR using mouse genomic DNA and primers: *Sox2*-F: GCCCA TGAAC GCCTT CATGG and T3-*Sox2*-R: aatta accct cacta aagg C ATGCT GATCA TGTCC CGGA. The *Gata3* probe template was described previously [15]. The Gata3 the transcription template was generated from the cDNA clone using SP6 and T7 primers.

### Three-dimensional reconstructions of histological sections

Comparisons of pouch morphology in 3D reconstructions from different embryos showed that pouch morphology is comparable between different embryos of the similar somite stage (data not shown). This similarity allowed us to make comparisons between reconstructions of different gene expression patterns. Digital images of serial sagittal paraffin sections from a single embryo were assembled into a three-dimensional (3D) image using the WinSurf 4.3 software. The gene expression positive areas and the third pharyngeal pouch endoderm were traced as separate objects.

### Immunofluorescence analysis of transcription factor expression

Dissected embryos or postnatal thymus tissue were treated differently for ISL1 and FOXG1 antibody staining. For ISL1 staining, E16.5 embryos were fixed in 4% PFA for 4 hours or postnatal thymus for 1.5 hr on ice. Fixed embryos or tissue were then washed three times in PBS and dehydrated through methanol series and embedded into paraffin blocks. 8 µm sections were cut on a Leica RM2155 microtome and de-waxed and rehydrated into water. Antigen retrieval was done by boiling the slides in AR buffer (10 mM Na_3_Citrate pH 6, 0.05% Tween20) for 30 minutes. After cooling down, slides were washed once with 0.05% PBST (0.05% Triton X-100) and blocked in 10% serum in PBST at room temperature for at least 30 minutes. Primary antibodies were mixed in 1% serum/PBST and incubated at 4°C overnight. After 3 PBST washes, secondary antibodies diluted in PBST were added and incubated at room temperature for 30 minutes. Slides were then washed and mounted in FluoroGel (EMS). Images were acquired using a Zeiss LSM510 META confocal imaging system.

For FOXG1 staining, E16.5 embryos were fixed in 4% PFA for 45 minute or postnatal thymus for 20 minutes on ice. Fixed embryos or tissue were then washed three times in PBS, then once in 5% sucrose/PBS and once in 15% sucrose/PBS before embedded and frozen in OCT compound (Sakura Tissue-Tek). 10 µm frozen sections were then cut on a Leica CM3050 S cryostat. The sections were blocked and incubated with primary and secondary antibodies as described for ISL1 staining.

The mouse anti-ISL monoclonal antibody was from the Developmental Studies Hybridoma Bank (clone#: 39.4D5, 1∶100). This ISL1 monoclonal was developed by Dr. Thomas Jessell and has a long track record of use in mice [51], [52], [53], [54], [55]. The other antibodies used in this work include rabbit anti-FOXG1 (Abcam, Cat#: ab18259, 1∶50) [56], [57], [58], [59], goat anti-*Foxn1* (Santa Cruz, G-20, 1∶200) [43], mouse anti-pan cytokeratin (Sigma, Cat#: C2931, 1∶800), rabbit anti-Keratin 5 (Covance, Cat#: PRB-160P, 1∶1000). Secondary antibodies were purchased from Invitrogen or Jackson Immunoresearch.
